# Correlation between cerebrospinal fluid abnormalities before ventriculoperitoneal shunt and postoperative intracranial infection in adult patients with hydrocephalus: A clinical study

**DOI:** 10.3389/fneur.2022.1023761

**Published:** 2023-01-24

**Authors:** Huan Zhang, Xiaozheng He, Linghai Xie, Hongbo Zhang, Xusheng Hou, Shizhong Zhang

**Affiliations:** ^1^Department of Neurosurgery, Affiliated Hospital No. 2 of Nantong University, First People's Hospital of Nantong City, Nantong, China; ^2^Guangdong Provincial Key Laboratory on Brain Function Repair and Regeneration, Department of Functional Neurosurgery, Zhujiang Hospital of Southern Medical University, Guangzhou, China

**Keywords:** ventriculoperitoneal shunt, cerebrospinal fluid, hydrocephalus, infection, risk factors

## Abstract

**Objective:**

To identify the relationship between preoperative cerebrospinal fluid (CSF) leukocyte, chloride, glucose, aspartate aminotransferase, lactate dehydrogenase, adenosine deaminase, lactic acid and protein levels and ventriculoperitoneal shunt infection.

**Methods:**

Records of 671 consecutive adult patients who underwent ventriculoperitoneal shunt surgery for the treatment of hydrocephalus at Zhujiang Hospital affiliated with Southern Medical University from January 2011 to March 2022 were reviewed. The patients were divided into infection and non-infection groups based on the presence of postoperative infection. For all patients, we analyzed age; sex; primary disease; preoperative CSF leukocyte, chloride, glucose, aspartate aminotransferase, lactate dehydrogenase, adenosine deaminase, lactic acid and protein levels; postoperative temperature; and postoperative infection.

**Results:**

A total of 397 patients were included, 28 (7.05%) of whom had an infection within 6 months of the operation and the remaining had no infection. There was no significant difference in age, sex, primary disease, leukocyte, chloride ion, aspartate aminotransferase, lactate dehydrogenase, adenosine deaminase and protein levels in CSF between infection group and non-infection group (*p* > 0.05). The postoperative infection rate of patients with CSF glucose < 2.8 mmol/L (*x*^2^ = 11.650, *p* = 0.001) and CSF lactic acid >2.8 mmol/L (*x*^2^ = 12.455, *p* < 0.001) was higher than that of patients with CSF glucose level ≥2.8 mmol/L and CSF lactic acid level in the range of (1–2.8) mmol/L, respectively, with statistical difference. Compared with the non-infection group, the level of CSF glucose (*t* = 4.113, *p* < 0.001) was significantly lower, and the level of CSF lactic acid (*t* = 6.651, *p* < 0.001) was significantly higher in the infection group. Multivariate logistic regression analysis showed that preoperative cerebrospinal fluid glucose < 2.8 mmol/L (OR = 3.911, 95% CI: 1.653~9.253, *p* = 0.002) and cerebrospinal fluid lactate >2.8 mmol/L (OR = 4.712, 95% CI: 1.892~11.734, *p* = 0.001) are risk factors for infection after ventriculoperitoneal shunt. ROC analysis revealed that the area under the curve (AUC) for CSF glucose and lactic acid level were 0.602 (95% CI: 0.492–0.713) and 0.818 (95% CI: 0.738–0.898), respectively. The infection group had higher rates of fever and body temperature on postoperative day 3–7 (*p* < 0.05).

**Conclusions:**

For adult hydrocephalus patients without clinical manifestations of intracranial infection but only with simple abnormality of cerebrospinal fluid, when the content of glucose in cerebrospinal fluid is < 2.8 mmol/L, and the content of lactic acid is >2.8 mmol/L, it is recommended to perform ventriculoperitoneal shunt after further improvement of cerebrospinal fluid indicators, otherwise, hasty operation will increase the postoperative infection rate. The postoperative fever rate of ventriculoperitoneal shunt surgery is high and the body temperature drops rapidly. If there is still fever after day 3 after surgery, whether there is intracranial infection should be considered.

## Introduction

Hydrocephalus refers to the imbalance between cerebrospinal fluid (CSF) production and absorption, and/or the obstruction of its pathways, resulting in disturbed CSF dynamics and the abnormal accumulation of excess CSF in either the ventricle or subarachnoid space, which then dilatates these areas (1, 2). Hydrocephalus commonly occurs in neurosurgical patients ranging from newborns to adults. It can be divided into primary and acquired hydrocephalus according to its etiology. Acquired hydrocephalus is known as a common sequelae in patients with post-hemorrhagic conditions, including brain trauma, intracranial hemorrhage, brain tumors, and intracranial infection, among others (3). The development of hydrocephalus is accompanied by a series of pathophysiological changes in periventricular structures and even the whole brain. Without timely and effective intervention, these changes can lead to serious consequences, such as serious neurological dysfunction, cognitive disturbances, and memory deficits. A ventriculoperitoneal shunt is the most common and effective method for the treatment of hydrocephalus (4–6). Early shunt surgery in patients leads to a better prognosis (2, 7, 8), therefore, early treatment is imperative.

Preoperative intracranial infection is an absolute contraindication for a ventriculoperitoneal shunt (9), however, it is difficult to diagnose, especially occult intracranial infections. CSF bacterial culture is the gold standard for diagnosis of intracranial infections in clinical practice, but the low positive rate and time-consuming procedure required for culture limits its use in practice. Clinically, most patients with hydrocephalus require prompt surgical treatment and thus, cannot wait for CSF bacterial culture results. For the vast majority of patients with negative CSF bacterial cultures, intracranial infection is assessed by CSF leukocyte count, chloride, glucose, aspartate aminotransferase, lactate dehydrogenase, adenosine deaminase, lactic acid, and protein levels in combination with clinical symptoms (fever, meningeal irritation sign). The clinical diagnosis of intracranial infection can be challenging due to issues such as the lack of a unified diagnostic standard as well as high false negative/positive rates (10, 11). However, because ventriculoperitoneal shunts are urgently needed for most hydrocephalus patients to improve symptoms, the presence of intracranial infection directly affects the patients' treatment plan and prognosis. Clinically, ventriculoperitoneal shunt operations are often still performed although the CSF leukocyte, chloride, glucose, aspartate aminotransferase, lactate dehydrogenase, adenosine deaminase, lactic acid and protein levels are not within normal range, and often do not result in postoperative intracranial infection. Therefore, the aim of this study was to identify the relationship between preoperative CSF leukocyte count, chloride, glucose, aspartate aminotransferase, lactate dehydrogenase, adenosine deaminase, lactic acid and protein levels and postoperative ventriculoperitoneal shunt infection.

## Subjects and methods

### Patients

We reviewed 671 consecutive adult patients who underwent ventriculoperitoneal shunt surgery for hydrocephalus treatment at Zhujiang Hospital affiliated with Southern Medical University from January 2011 to March 2022.

### Inclusion and exclusion criteria

The inclusion criteria for this study were stable vital signs, absence of fever and neck stiffness, no previous operations, preoperative lumbar puncture results, negative CSF bacterial culture, normal preoperative blood levels of leukocyte, glucose, chloride, aspartate aminotransferase, lactate dehydrogenase, adenosine deaminase, lactic acid and protein, operative time within 2 h, and at least a 6-month follow-up. The exclusion criteria were as follows: no cerebrospinal fluid examination within 48 h before surgery; abnormal preoperative blood levels of leukocytes, glucose, chloride, aspartate aminotransferase, lactate dehydrogenase, adenosine deaminase, lactic acid and protein; clear history of intracranial infection within 3 months before the surgery; positive CSF bacteria culture within 3 months before the surgery; preoperative procalcitonin >0.05; preoperative fever; positive meningeal irritation sign; previous history of a ventriculoperitoneal shunt; operative time over 2 h; follow-up time of < 6 months; and a diabetes diagnosis. Ultimately, we enrolled 397 patients who met the inclusion criteria.

### Treatment and research methods

All patients were treated with prophylactic antibiotics half an hour before ventriculoperitoneal shunt surgery and symptomatic treatment after surgery. All patients completed lumbar puncture and CSF collection within 48 h before surgery, and the laboratory department of Zhujiang Hospital affiliated with Southern Medical University completed routine and biochemical examination of CSF. Collect and compare the general clinical data, preoperative cerebrospinal fluid test results and postoperative temperature of patients in the infection group and non-infection group, and determine the correlation between preoperative routine and biochemical indicators of cerebrospinal fluid and intracranial infection after ventriculoperitoneal shunt.

### Clinical data collection

We retrospectively analyzed the following data: age; sex; protopathy; and preoperative CSF leukocyte, chloride, glucose, and protein levels. The normal ranges for CSF were as follows: white blood cell (WBC) count, (0–8) × 10^6^/L; chloride, (120–130) mmol/L; glucose, (2.8–4.5) mmol/L; aspartate aminotransferase, (5–20) mmol/L; lactate dehydrogenase, (3–40) U/L; adenosine deaminase, (0–8) U/L; lactic acid, (1–2.8) mmol/L and protein, (150–450) mg/L. The highest body temperature was recorded on the day of surgery and 1–7 days after surgery. A temperature higher than 37.3°C was defined as a fever.

### Diagnostic criteria of intracranial infection

Current Centers for Disease Control and Prevention/National Healthcare Safety Network criteria for diagnosis of meningitis were used (12). Intracranial infection must meet at least one of the following criteria: (1) Patient has organism (s) identified from brain tissue or dura by a culture or non-culture based microbiologic testing method which is performed for purposes of clinical diagnosis or treatment, for example, not Active Surveillance Culture/Testing (ASC/AST). (2) Patient has an abscess or evidence of intracranial infection on gross anatomic or histopathologic exam. (3) Patient has at least two of the following signs or symptoms: headache, dizziness, fever (>38.0°C), localizing neurologic signs, changing level of consciousness, or confusion. And at least one of the following: (a) organism (s) seen on microscopic examination of brain or abscess tissue obtained by needle aspiration or during an invasive procedure or autopsy. (b) imaging test evidence suggestive of infection (for example, ultrasound, CT scan, MRI, radionuclide brain scan, or arteriogram), which if equivocal is supported by clinical correlation, specifically, physician documentation of antimicrobial treatment for intracranial infection. c. diagnostic single antibody titer (IgM) or 4-fold increase in paired sera (IgG) for organism. Meningitis or ventriculitis must meet at least one of the following criteria: (1) Patient has organism (s) identified from cerebrospinal fluid (CSF) by a culture or non-culture based microbiologic testing method which is performed for purposes of clinical diagnosis or treatment for example, not Active Surveillance Culture/Testing (ASC/AST). (2) Patient has at least two of the following: (i) fever (>38.0°C) or headache (Note: Elements of “*i*” alone may not be used to meet the two required elements), (ii) meningeal sign (s), and (iii) cranial nerve sign (s) And at least one of the following: (a) increased white cells, elevated protein, and decreased glucose in CSF (per reporting laboratory's reference range). (b) organism (s) seen on Gram stain of CSF. (c) organism (s) identified from blood by a culture or non-culture based microbiologic testing method which is performed for purposes of clinical diagnosis or treatment, for example, not Active Surveillance Culture/Testing (ASC/AST). (d) diagnostic single antibody titer (IgM) or 4-fold increase in paired sera (IgG) for organism.

### Statistical analysis

SPSS statistical software (Version 25.0. Armonk, NY: IBM Corp) was used for all data analyses. First, single factor analysis was performed on the data. Continuous variables are presented as mean ± standard deviation and range, and categorical variables are presented as the number of cases. To compare baseline variables, the chi-square and Fisher's exact tests were used for categorical variables, and a Student's *t*-test was used for continuous variables. A *p*-value < 0.05 was considered statistically significant. A logistic stepwise regression was used to analyze the multiple risk factors for infection after a ventriculoperitoneal shunt. The probability of stepwise entry was 0.05 and the probability of removal was 0.10. The ROC curve was used to analyze how well each factor predicted postoperative infection.

## Results

### Demographics and patient characteristics

A total of 397 patients were included; 28 (7.05%) developed an infection within 6 months after the operation and the remaining 369 (92.95%) did not develop an infection. A summary of patient demographics and clinical characteristics is shown in Tables 1, **3**. Of the 397 patients, 226 were males and 171 females and the average age was 49.80 ± 0.75 years. There were 260 cases of cerebral hemorrhage, 73 cases of brain tumor, 20 cases of idiopathic normal pressure hydrocephalus, 12 cases of tuberculous meningitis, 14 cases of cerebral infarction, 11 cases of cerebral cyst, and 7 cases of others. Of the 28 infected patients, all infections occurred within 6 months after surgery, including 10 cases (35.7%) 1 week, 2 cases (7.1%) 2 weeks, 10 cases (35.7%) 1 month, 3 cases (10.7%) 2 months, 2 cases (7.1%) 4 months, and 1 case (3.6%) six months after surgery (Table 2).

**Table 1:** Single factor analysis of the difference between the two groups in gender, primary disease and CSF parameters.

**Variable**	**Non-infection group (*n* = 369) *n* (%)**	**Infection group (*n* = 28) *n* (%)**	**Infection rate (%)**	**Chi-square**	***p*-value**
Gender				0.001	0.981
Male	210 (56.9)	16 (57.1)	7.1%		
Female	159 (43.1)	12 (42.9)	7.0%		
Primary disease				2.939	0.816
Cerebral hemorrhage	243 (65.9)	17 (60.7)	6.5%		
Brain tumor	67 (18.2)	6 (21.4)	8.2%		
Idiopathic normal pressure hydrocephalus	19 (5.1)	1 (3.6)	5.0%		
Tuberculous meningitis	11 (3.0)	1 (3.6)	8.3%		
Cerebral infarction	13 (3.5)	1 (3.6)	7.1%		
Cerebral cyst	9 (2.4)	2 (7.1)	18.2%		
Others	7 (1.9)	0 (0)	0.0%		
CSF leukocyte level				0.684	0.408
(0–8) × 10^6^/L	252 (68.3)	17 (60.7)	6.3%		
>8 × 10^6^/L	117 (31.7)	11 (39.3)	8.6%		
CSF chloride level				0.821	0.663
< 120 mmol/L	114 (30.9)	8 (28.6)	6.6%		
(120–130) mmol/L	228 (61.8)	19 (67.9)	7.7%		
>130 mmol/L	27 (7.3)	1 (3.6)	3.6%		
CSF glucose level				11.650	0.001
< 2.8 mmol/L	62 (16.8)	12 (60.7)	16.2%		
≥2.8 mmol/L	307 (76.4)	16 (39.3)	5.0%		
CSF aspartate aminotransferase level				< 0.001	>0.999
(5–20) U/L	304 (82.4)	23 (82.1)	7.0%		
>20 U/L	65 (17.6)	5 (17.9)	7.1%		
CSF lactate dehydrogenase level				1.520	0.218
(3–40) U/L	299 (81.0)	20 (71.4)	6.3%		
>40 U/L	70 (19.0)	8 (28.6)	10.3%		
CSF adenosine deaminase level				< 0.001	>0.999
(0–8) U/L	330 (89.4)	25 (89.3)	7.0%		
>8 U/L	39 (10.6)	3 (10.7)	7.1%		
CSF lactic acid level				12.455	< 0.001
(1–2.8) mmol/L	329 (89.2)	18 (64.3)	5.2%		
>2.8 mmol/L	40 (10.8)	10 (35.7)	20%		
CSF protein level				2.245	0.325
< 450 mg/L	176 (47.7)	12 (42.9)	6.4%		
(450–1,000) mg/L	129 (35.0)	8 (28.6)	5.8%		
>1,000 mg/L	64 (17.3)	8 (28.6)	11.1%		

**Table 2:** Time distribution of postoperative infection.

**Infection time after surgery**	**Case**	**Proportion (%)**
1 week	10	35.7
2 weeks	2	7.1
1 month	10	35.7
2 months	3	10.7
4 months	2	7.1
6 months	1	3.6
Total	28	100

### Single factor analysis of infectious factors after ventriculoperitoneal shunt operation

The patients were divided into non-infection and infection groups, based on the presence of infection after ventriculoperitoneal shunt operation. Chi-square and Fisher exact tests were used to analyze and compare the differences in sex; age; protopathy; and preoperative CSF leukocyte, chloride, glucose, aspartate aminotransferase, lactate dehydrogenase, adenosine deaminase, lactic acid and protein levels between the two groups. There were 28 cases of infection after operation, yielding an infection rate of 7.05% (28/397). The postoperative infection rate of patients with CSF glucose level < 2.8 mmol/L (*x*^2^ = 11.650, *p* = 0.001) and lactic acid level >2.8 mmol/L (*x*^2^ = 12.455, *p* < 0.001) was higher than that of patients with CSF glucose level ≥2.8 mmol/L and CSF lactic acid level in the range of (1–2.8) mmol/L, respectively, with statistical difference. There was no significant difference in sex, primary disease, CSF leukocyte, chloride, aspartate aminotransferase, lactate dehydrogenase, adenosine deaminase, and protein levels between infected group and non-infected group (all *p* > 0.05; Table 1).

A Student's *t*-test was used to analyze and compare the differences in age and preoperative CSF leukocyte, chloride, glucose, aspartate aminotransferase, lactate dehydrogenase, adenosine deaminase, lactic acid and protein levels between the non-infection and infection groups. Compared with the non-infection group, the level of CSF glucose (*t* = 4.113, *p* < 0.001) was significantly lower, and the level of CSF lactic acid (*t* = 6.651, *p* < 0.001) was significantly higher in the infection group. There was no significant difference in age, CSF leukocyte level, CSF chloride level, CSF aspartate aminotransferase level, CSF lactate dehydrogenase level, CSF adenosine deaminase level and CSF protein level between infected and non-infected patients (all *p* > 0.05; Table 3).

**Table 3:** Single factor analysis of the difference between the two groups in age and CSF parameters.

**Variable**	**Non-infection group (Mean ±SD)**	**Infection group (Mean ±SD)**	***T*-value**	***p*-value**
Age (years)	49.87 ± 15.18	48.52 ± 10.26	0.634	0.530
CSF leukocyte level (× 10^6^/L)	14.14 ± 67.56	19.39 ± 44.17	0.404	0.686
CSF chloride level (mmol/L)	121.84 ± 10.14	123.56 ± 5.94	0.885	0.376
CSF glucose level (mmol/L)	3.40 ± 0.88	2.71 ± 0.49	4.113	< 0.001
CSF aspartate aminotransferase level (U/L)	14.07 ± 20.02	13.14 ± 8.94	0.243	0.808
CSF lactate dehydrogenase level (U/L)	29.65 ± 50.98	24.85 ± 14.25	0.496	0.620
CSF adenosine deaminase level (U/L)	2.49 ± 3.41	2.34 ± 2.63	0.231	0.817
CSF lactic acid level (mmol/L)	2.08 ± 0.70	3.00 ± 0.77	6.651	< 0.001
CSF protein level (mg/L)	577.36 ± 373.55	693.60 ± 407.23	1.577	0.116

### Multivariate analysis of infectious factors after ventriculoperitoneal shunt operation

**Figure 1:**
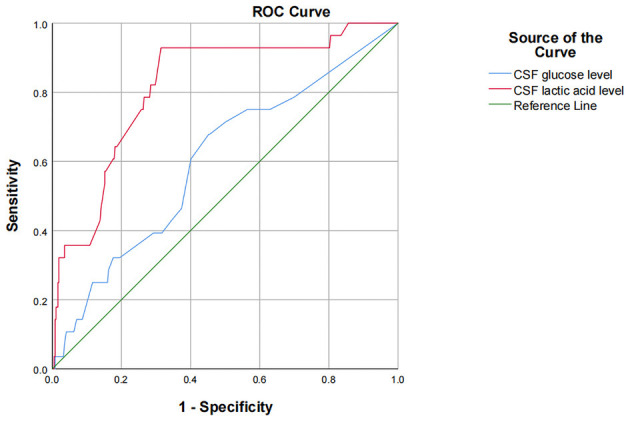
Roc curve of cerebrospinal fluid glucose and lactate levels.

The results of univariate analysis showed that there were statistically significant differences in preoperative CSF glucose and lactic acid level. Because of the interaction among preoperative leukocyte, chloride, glucose, aspartate aminotransferase, lactate dehydrogenase, adenosine deaminase, lactic acid and protein levels, CSF indicators were all introduced into binary logistic stepwise regression analyses as independent variables. Multivariate logistic regression analysis showed that preoperative CSF glucose < 2.8 mmol/L and CSF lactate >2.8 mmol/L are risk factors for infection after ventriculoperitoneal shunt. Compared with patients with preoperative glucose level in cerebrospinal fluid ≥2.8 mmol/L, patients with glucose level < 2.8 mmol/L had a 2.911-fold increased probability of intracranial infection after surgery (OR = 3.911, 95% CI: 1.653~9.253, *p* = 0.002); Compared with patients with preoperative cerebrospinal fluid lactic acid level of 1–2.8 mmol/L, patients with lactic acid level >2.8 mmol/L had a 3.712-fold increased probability of intracranial infection after surgery (OR = 4.712, 95% CI: 1.892~11.734, *p* = 0.001; Table 4). Using ROC to understand the effect of these two factors, we found that the area under the curve (AUC) for CSF glucose and lactic acid levels were 0.602 (95% CI: 0.492–0.713) and 0.818 (95% CI: 0.738–0.898), respectively (Figure 1).

**Table 4:** Multifactor analysis of postoperative infection caused by cerebrospinal fluid parameters before ventriculoperitoneal shunt.

**CSF parameters**	***B***	**SE**	**Wald**	**OR**	**95% CI**	***p*-value**
**CSF leukocyte level (**×**10**^6^**/L)**						
0–8^*^						
>8	0.152	0.445	0.116	1.164	0.487~2.781	0.733
**CSF chloride level (mmol/L)**						
< 120^*^						
120–130	0.364	0.467	0.607	1.439	0.576~3.594	0.436
>130	−0.495	1.108	0.199	0.610	0.070~5.349	0.655
**CSF glucose level (mmol/L)**						
< 2.8	1.364	0.439	9.634	3.911	1.653~9.253	0.002
≥2.8^*^						
**CSF aspartate aminotransferase level (U/L)**						
5–20^*^						
>20	−0.376	0.614	0.376	0.686	0.206~2.286	0.540
**CSF lactate dehydrogenase level (U/L)**						
3–40^*^						
>40	0.371	0.524	0.501	1.449	0.519~4.048	0.479
**CSF adenosine deaminase level (U/L)**						
0–8^*^						
>8	−0.103	0.699	0.022	0.902	0.229~3.551	0.883
**CSF lactic acid level (mmol/L)**						
1–2.8^*^						
>2.8	1.550	0.466	11.088	4.712	1.892~11.734	0.001
**CSF protein level (mg/L)**						
< 450^*^						
450–1,000	−0.019	0.500	0.001	0.981	0.368~2.614	0.970
>1,000	0.082	0.547	0.022	1.085	0.371~3.171	0.881
Constant	−3.583	0.527	46.207	0.028		0

^*^Reference group.

### The relationship between postoperative body temperature and infection

Postoperative fever of the 397 patients who underwent surgery are shown for different times post-operation in Table 5. Most of the patients had no fever on the day of the surgery, with only 10.6% of patients experiencing fever. The proportion of the patients with a fever was highest on the first day after the surgery, with a rate of 54.9%, though this fever typically gradually decreased over time.

**Table 5:** Statistical table of postoperative fever.

**Group**	**The day of surgery**	**The 1st day after surgery**	**The 2nd day after surgery**	**The 3rd day after surgery**	**The 4th day after surgery**	**The 5th day after surgery**	**The 6th day after surgery**	**The 7th day after surgery**
Fever	42 (10.6%)	218 (54.9%)	174 (43.8%)	158 (39.8%)	141 (35.5%)	109 (27.5%)	74 (18.6%)	72 (18.1%)
No fever	355 (89.4%)	179 (45.1%)	223 (56.2%)	239 (60.2%)	256 (64.5%)	288 (72.5%)	323 (81.4%)	325 (81.9%)

There was no statistically significant difference in the fever rate on the day of surgery, the 1st and the 2nd day after surgery between the non-infection and infection groups (all *p* > 0.05). However, compared to the non-infection group, the fever rate of the infection group was significantly higher on postoperative day 3–7 (all *p* < 0.05; Table 6).

**Table 6:** Comparison of postoperative fever between the non-infection and infection groups.

**Postoperative body temperature**	**Non-infection group (*n* = 247) *n* (%)**	**Infection group (*n* = 25) *n* (%)**	**Chi-square**	***p*-value**
The day of surgery			0.000	>0.999
No fever	330 (89.4)	25 (89.3)		
Fever	39 (10.6)	2 (10.7)		
The 1st day after surgery			0.410	0.522
No fever	168 (45.5)	11 (39.3)		
Fever	201 (54.5)	17 (60.7)		
The 2nd day after surgery			0.083	0.774
No fever	208 (56.4)	15 (53.6)		
Fever	161 (43.6)	13 (46.4)		
The 3rd day after surgery			7.539	0.006
No fever	229 (62.1)	10 (35.7)		
Fever	140 (37.9)	18 (64.3)		
The 4th day after surgery			8.352	0.004
No fever	245 (66.4)	11 (39.3)		
Fever	124 (33.6)	17 (60.7)		
The 5th day after surgery			7.687	0.006
No fever	274 (74.3)	14 (50.0)		
Fever	95 (25.7)	14 (50.0)		
The 6th day after surgery			24.239	< 0.001
No fever	310 (84.0)	13 (46.4)		
Fever	59 (16.0)	15 (53.6)		
The 7th day after surgery			16.242	< 0.001
No fever	310 (82.6%)	15 (56%)		
Fever	59 (17.4%)	13 (44%)		

Compared to the non-infection group, patients in the infection group had significantly higher body temperatures on postoperative day 3–7 (all *p* < 0.05). However, there was not a significant difference in body temperature between the non-infection and infection groups on the day of surgery, the 1st and the 2nd days after surgery (all *p* > 0.05). The body temperature of the patients in the non-infection group peaked on the first day after surgery and then gradually decreased, dropping below 37.3°C on the 4th day. The body temperature of the patients in the infection group also reached the peak on the first day after surgery, but the subsequent drop was not obvious, and it was always above 37.3°C on the 2nd to 7th days after surgery (Table 7).

**Table 7:** Comparison of postoperative body temperature between the non-infection and infection groups.

**Group**	**Temperature on the day of surgery (°C)**	**Temperature on the 1st day after surgery (°C)**	**Temperature on the 2nd day after surgery (°C)**	**Temperature on the 3rd day after surgery (°C)**	**Temperature on the 4th day after surgery (°C)**	**Temperature on the 5th day after surgery (°C)**	**Temperature on the 6th day after surgery (°C)**	**Temperature on the 7th day after surgery (°C)**
Non-infection group (Mean ± SD)	36.83 ± 0.59	37.64 ± 0.73	37.56 ± 0.60	37.32 ± 0.83	37.27 ± 0.43	37.28 ± 0.64	37.13 ± 0.44	37.13 ± 0.60
Infection group (Mean ± SD)	36.88 ± 0.56	37.98 ± 0.99	37.81 ± 0.70	37.79 ± 0.75	37.79 ± 0.57	37.80 ± 0.87	37.81 ± 0.91	37.81 ± 0.92
*T*-value	0.439	1.739	1.832	2.872	4.692	3.091	3.946	3.885
*p*-value	0.661	0.093	0.077	0.004	< 0.001	0.004	< 0.001	0.001

## Discussion

Many patients with hydrocephalus secondary to cerebral hemorrhage after brain trauma, spontaneous cerebral hemorrhage, brain tumors, cerebral infarction and other diseases, often due to the impact of the primary disease or the impact of surgical procedures taken to treat the primary disease, leading to the occurrence of aseptic inflammation in the ventricular system, resulting in long-term abnormality of routine and biochemical parameters of cerebrospinal fluid (10, 11). A few patients with idiopathic hydrocephalus also have this situation. However, patients with such hydrocephalus often need to be checked for improved CSF indicators through repeated lumbar punctures, external lumbar cistern drainage, and external lateral ventricle drainage, among other, thus missing the best time for operation, ultimately delaying or even aggravating the condition (2, 13, 14). Rammos et al. (15) reported that the average time for external ventricular drainage before the conversion to internal drainage was 14.1 days, though this could sometime last as long as 45 days. Bota et al. (16) reported that the infection rate associated with extraventricular drainage was 0–22%, and the infection rate showed a straight increase between days 3 and 9 after operation. In hydrocephalus, if the central nervous system becomes involved in infection, the treatment becomes very difficult. There are few reports on the safe range of routine and biochemical indicators of cerebrospinal fluid in hydrocephalus patients undergoing ventriculoperitoneal shunt, and most clinicians choose the time of ventriculoperitoneal shunt based on experience. Therefore, this study retrospectively analyzed the clinical characteristics of 397 patients with hydrocephalus and attempted to summarize the relationship between abnormal preoperative CSF leukocyte, chloride, glucose, aspartate aminotransferase, lactate dehydrogenase, adenosine deaminase, lactic acid and protein levels and postoperative infection after a ventriculoperitoneal shunt to guide appropriate operation time for hydrocephalus patients with abnormal CSF.

The incidence of infection after a ventriculoperitoneal shunt can vary in prevalence, ranging from about 3–12% (16). In our study, 28 of the total 397 patients (7.05%) had postoperative infection, consistent with the reported postoperative infection rate in most international clinical centers. In our study, there was no difference in the CSF leukocyte, chloride, aspartate aminotransferase, lactate dehydrogenase, adenosine deaminase and protein levels between the non-infection and infection groups; however, there were significant differences in CSF glucose and lactic acid levels. Therefore, this study demonstrates that abnormal CSF leukocyte, chloride, aspartate aminotransferase, lactate dehydrogenase, adenosine deaminase and protein levels do not increase the infection rate after ventriculoperitoneal shunt surgery. Due to the presence of aseptic inflammation and the interference of CSF with various indicators, CSF leukocytes, chloride, aspartate aminotransferase, lactate dehydrogenase, adenosine deaminase and proteins could not accurately reflect intracranial infection. Therefore, for hydrocephalus patients with no fever, negative meningeal stimulation, and a negative CSF bacterial culture, if only CSF leucocyte, chloride, aspartate aminotransferase, lactate dehydrogenase, adenosine deaminase and protein levels are abnormal, the ventriculoperitoneal shunt should be performed as early as possible, as avoiding external drainage increases the risk of infection and delays the timing of shunt surgery. The physical properties of normal CSF are similar to that of water. Brydon et al. (17, 18) studied CSF of 126 patients with hydrocephalus of different etiologies and found that its physical properties do not change significantly and are still similar to those of water. Rammos et al. (15) reported that CSF protein levels and RBC counts do not have adverse effects on shunt survival, and therefore, increased protein or RBC counts should not delay the transition of ventriculoperitoneal shunt into ventriculoperitoneal shunt, consistent with the findings of this study.

This study showed that CSF glucose below the normal range and CSF lactic acid above the normal range increase the postoperative infection rate. Moreover, multivariate logistic stepwise regression analysis indicates that preoperative CSF glucose < 2.8 mmol/L and CSF lactate >2.8 mmol/L are risk factors for infection after ventriculoperitoneal shunt. Compared with patients with preoperative glucose level in cerebrospinal fluid ≥2.8 mmol/L, patients with glucose level < 2.8 mmol/L had a 2.911-fold increased probability of intracranial infection after surgery (OR = 3.911, 95% CI: 1.653~9.253, *p* = 0.002); Compared with patients with preoperative cerebrospinal fluid lactic acid level of 1–2.8 mmol/L, patients with lactic acid level >2.8 mmol/L had a 3.712-fold increased probability of intracranial infection after surgery (OR = 4.712, 95% CI: 1.892~11.734, *p* = 0.001). Using ROC to understand the effect of these two factors, we found that the area under the curve (AUC) for CSF glucose and lactic acid levels were 0.602 (95% CI: 0.492–0.713) and 0.818 (95% CI: 0.738–0.898), respectively. One possible explanation for this is that it may be difficult to culture pathogenic bacteria from patients with low CSF glucose level or high CSF lactate level due to preoperative empirical, broad-spectrum antibiotic use, even if there is actually an insidious intracranial infection, ultimately leading to a postoperative shunt infection. When hydrocephalus patients with occult intracranial infection are implanted into the ventriculoperitoneal shunt system, the bacteria in the ventricles attach to and proliferate on the ventriculoperitoneal shunt system, and form bacterial biofilms that are difficult to kill by antibiotics (19). Finally, the patients show obvious intracranial infection. CSF glucose content accounts for 1/2~2/3 of the blood glucose value, mainly because glucose in peripheral serum is transported into cerebrospinal fluid through glucose transporter. In patients with intracranial infection, CSF glucose levels decrease, though the mechanism by which this change occurs in the central nervous system is not fully understood. Intracranial infection increases glycolysis and thus reduces the glucose in CSF. Intracranial infection also affects the function of glucose transporters, thereby reducing the transport of glucose from blood to CSF. The content of lactic acid in cerebrospinal fluid is very low. Most of it is the product of anaerobic fermentation of glucose in cerebrospinal fluid, which is not easily affected by the level of lactic acid in peripheral blood, so lactic acid can accurately reflect the metabolism in the brain (20). The change in CSF glucose and lactic acid content may be more sensitive and specific to the diagnosis of occult intracranial infection. Mrria et al. (21) demonstrated that low glucose content in CSF is of high diagnostic and predictive value for intracranial infections caused by specific bacteria. The change in CSF glucose content is more effective than CSF WBC count for detecting intracranial infection. A prospective study by Pedro Grille et al. (22) showed that CSF glucose and lactic acid had the best diagnostic accuracy for ventriculostomy-related infection, with an AUC of 0.951 and 0.900 (*p* = 0.001), respectively. CSF WBC count and protein level were not able to diagnose ventriculostomy-related infection better than CSF glucose and lactic acid. Tavares et al. (23) conducted a prospective study on 28 neurosurgical patients and found that CSF glucose level is better than leukocyte count and protein index for the diagnosis of intracranial infection. Eduardo et al. (10) found that the sensitivity of 3 mmol/L CSF lactic acid to bacterial meningitis was 95%, the specificity was 94%, and the negative predictive value was 99.3%. A prospective study conducted by Maskin et al. (24) also drew a similar conclusion that the increase of cerebrospinal fluid lactic acid has a better predictive value in the diagnosis of intracranial infection than the increase of the number of cells in cerebrospinal fluid. When the level of CSF lactic acid is ≥4 mmol/L, the sensitivity and specificity of the diagnosis of intracranial infection are 97 and 78% respectively. Two other meta-analyses pointed out that the accuracy of cerebrospinal fluid lactic acid in the diagnosis of intracranial infection was better than other indicators in cerebrospinal fluid (25, 26). Therefore, the content of glucose and lactic acid in cerebrospinal fluid has high diagnostic value for intracranial infection. In this study, when cerebrospinal fluid glucose < 2.8 mmol/L, the infection rate was as high as 16.2%, while when cerebrospinal fluid lactic acid >2.8 mmol/L, the infection rate was as high as 20%, both of which were significantly higher than the infection rate of this study of 7.05%. Therefore, it is reasonable to believe that such patients have latent intracranial infection before surgery, leading to infection after ventriculoperitoneal shunt surgery. To sum up, for patients with hydrocephalus without clinical manifestations of intracranial infection such as fever and positive meningeal irritation sign, if their CSF glucose level is < 2.8 mmol/L and CSF lactic acid is >2.8 mmol/L, it is recommended to conduct further examination and treatment for the cerebrospinal fluid, and the ventriculoperitoneal shunt should be considered after their CSF glucose and lactic acid content are improved to the normal range.

Many health centers have studied infection risk factors after shunt surgery to better predict the risk of infection and take preventive measures. These risk factors include premature infants, young patients, specific causes of hydrocephalus, postoperative CSF leakage, duration of surgery (9, 27, 28), and previous stream operations, even the largest risk factors (29–31). Therefore, for premature infants, young patients, and patients with previous histories of shunt surgery, more attention should be given to ensure aseptic operating conditions and to reduce the exposure and contact of the shunt system during surgery. Additional measures could include wearing double gloves (28), soaking the shunt tube with antibiotics (32, 33), and limiting the use of perioperative antibiotics (34).

Ventriculoperitoneal shunt surgery is a common neurosurgery and postoperative infection is one of the most serious complications, often leading to failed surgery and sometimes even requiring the removal of the shunt. Early diagnosis and treatment of postoperative infection may prevent the failure of shunt surgery, so it is very important. Fever is one of the main manifestations of infection, but early postoperative fever is also common in neurosurgery (35–37), making infection difficult to distinguish in patients. Understanding fever trends are important, as the body temperature of non-infected patients tends to decrease over time. In this study, the body temperature of patients without infection after ventriculoperitoneal shunt surgery presented with a single peak in fever the first day after surgery, which then gradually decreased, reaching lower than 37.3°C on the 4th days, and normalizing thereafter. However, the body temperature of infected patients did not decrease significantly over time. Though their temperature also peaked on the first day after surgery, it remained higher than 37.3°C from day 2–7 after surgery. The body temperature of the infected patients was higher than those without infection from day 3–7 after surgery. Unsurprisingly, the fever rate of the infection group from day 3–7 after surgery was significantly higher than that of the non-infection group (64.3 vs. 37.9, 60.7 vs. 33.6, 50.0 vs. 25.7, 53.6 vs. 16.0, 44.0 vs. 17.4%, respectively). The common cause of fever after neurosurgery is usually heat absorption, therefore a physiological fever caused by the liquefaction and necrosis of the tissue and the absorption of this necrotic tissue into the blood, activating neutrophils, eosinophils, and mononuclear macrophage systems to release endogenous pyrogen. Although the trauma of ventriculoperitoneal shunt surgery is small, it changes CSF circulation. Additionally, the shunt system is implanted into the human body, which is actually an indoor surgery. If small amounts of blood or surgical materials are mixed with CSF during the surgery, postoperative fever can occur. As the ventriculoperitoneal shunt drainage accelerates, the CSF circulation increases, meaning that the fever should also subside faster. Thus, fever after ventriculoperitoneal shunt surgery typically subsides faster than other neurosurgeries; the body temperature is generally within normal range by the third day after surgery. If a fever persists after the third day, then the clinician should be notified. When the fever cannot otherwise be explained, a lumbar puncture should be considered so that the CSF can be tested. When combined with other clinical manifestations, such as positive meningeal stimulation, aggravation of consciousness, headache, vomiting, and redness and swelling in the shunt, the prospect of an intracranial infection should be given serious consideration and anti-infection therapies should be utilized.

Post-ventriculoperitoneal shunt infections generally occur within 6 months after surgery (38, 39), with ~70% diagnosed within a month after surgery and more than 90% diagnosed within 6 months (40). In this study, infections occurred in all 28 infected patients within 6 months after surgery; 10 patients (35.7%) were infected 1 week, 2 patients (7.1%) were infected 2 weeks, 10 patients (35.7%) were infected 1 month, 3 patients (10.7%) were infected 2 months, two patients (7.1%) were infected 4 months, and 1 patient (3.6%) was infected 6 months after surgery, consistent with the infection time reported in the literature. The early onset of infection could be due to the presence of skin or related pathogens during the operation, which could be results from a lack of strict aseptic operating conditions and contamination of the shunt system (41). It could also be due to abnormal CSF before surgery indicating the presence of an occult intracranial infection, leading to the outbreak of an intracranial infection in the early postoperative period. Ventriculoperitoneal shunt infection is the most serious complication of ventriculoperitoneal shunt surgery for hydrocephalus and can prolong the hospital stay, cost more money, and even lead to death. Therefore, once intracranial infection is considered, standardized treatment should be promptly conducted. Of the 25 cases of infection in our study, one case was treated only with antibiotics; 10 cases were treated with antibiotics first and shunts were then removed and ventricular drainage was performed because of poor infection control; and 14 cases were treated by removing the shunts, using extraventricular drainage, and giving systemic antibiotics. The one patient who was only treated with antibiotics died; however, the intracranial infection was resolved in all other cases. During shunt infection, pathogenic bacteria proliferate on the material of the shunt to form a dense “biofilm” that protects the bacteria from being killed by antibodies, leukocytes, and antibiotics (42). Therefore, it is very difficult to treat shunt infection with systemic or intraventricular antibiotics, necessitating the removal of shunt tube (43, 44). Schreffler et al. (45) concluded that the best treatment for intracranial infection is removal of the shunt, external ventricular drainage, and application of antibiotics.

This study is a single-center retrospective study with inherent limitations, meaning that all results need to be verified with multi-center, prospective studies. Ventriculoperitoneal shunt shunts the cerebrospinal fluid in the lateral ventricles, but the cerebrospinal fluid circulation of patients with hydrocephalus is affected, so the cerebrospinal fluid obtained by lumbar puncture may not represent the nature of the cerebrospinal fluid in the lateral ventricles. Therefore, the difference and correlation between cerebrospinal fluid obtained by lumbar puncture and cerebrospinal fluid in lateral ventricle need further prospective study.

## Conclusions

For adult hydrocephalus patients without clinical manifestations of intracranial infection but only with simple abnormality of cerebrospinal fluid, when the content of glucose in cerebrospinal fluid is < 2.8 mmol/L, and the content of lactic acid is more than 2.8 mmol/L, it is recommended to perform ventriculoperitoneal shunt after further improvement of cerebrospinal fluid indicators, otherwise, hasty operation will increase the postoperative infection rate. The postoperative fever rate of ventriculoperitoneal shunt surgery is high and the body temperature drops rapidly. If there is still fever after day 3 after surgery, whether there is intracranial infection should be considered.

## Data availability statement

The raw data supporting the conclusions of this article will be made available by the authors, without undue reservation.

## Ethics statement

The studies involving human participants were reviewed and approved by the Ethics Committee of Zhujiang Hospital of Southern Medical University. Written informed consent from the patients/participants or patients/participants' legal guardian/next of kin was not required to participate in this study in accordance with the national legislation and the institutional requirements.

## Author contributions

HuZ: conceptualization, methodology, software, validation, formal analysis, investigation, data curation, writing—original draft, and writing—reviewing and editing. XHe: conceptualization, methodology, formal analysis, and writing—reviewing and editing. LX: investigation and data curation. HoZ and XHo: writing—reviewing and editing. SZ: conceptualization, methodology, resources, writing—reviewing and editing, supervision, and project administration. All authors contributed to the article and approved the submitted version.
